# Vagotomy accelerates the onset of symptoms during early disease progression and worsens joint-level pathogenesis in a male rat model of chronic knee osteoarthritis

**DOI:** 10.1016/j.ocarto.2024.100467

**Published:** 2024-04-08

**Authors:** Carlos J. Cruz, Taylor D. Yeater, Jacob L. Griffith, Kyle D. Allen

**Affiliations:** a J. Crayton Pruitt Family Department of Biomedical Engineering, University of Florida, Gainesville, FL, USA; b Department of Orthopaedic Surgery and Sports Medicine, College of Medicine, University of Florida, Gainesville, FL, USA; c Pain Research and Intervention Center of Excellence, University of Florida, Gainesville, FL, USA

**Keywords:** Osteoarthritis, Autonomic nervous system, Vagotomy, Brain-joint axis, Rat model

## Abstract

**Objective:**

Low vagal tone is common in osteoarthritis (OA) comorbidities and results in greater peripheral inflammation. Characterizing vagal tone's role in OA pathogenesis may offer insights into OA's influences beyond the articular joint. We hypothesized that low vagal tone would accelerate onset of OA-related gait changes and worsen joint damage in a rat knee OA model.

**Methods:**

Knee OA was induced in male Sprague Dawley rats by transecting the medial collateral ligament and medial meniscus. Then, left cervical vagus nerve transection (VGX, n ​= ​9) or sham VGX (non-VGX, n ​= ​6) was performed. Gait and tactile sensitivity were assessed at baseline and across 12 weeks, with histology and systemic inflammation evaluated at endpoint.

**Results:**

At week 4, VGX animals showed limping gait characteristics through shifted stance times from their OA to non-OA limb (p ​= ​0.055; stance time imbalance ​= ​1.6 ​± ​1.6%) and shifted foot strike locations (p ​< ​0.001; spatial symmetry ​= ​48.4 ​± ​0.835%), while non-VGX animals walked with a balanced and symmetric gait. Also at week 4, while VGX animals had a mechanical sensitivity (50% withdrawal threshold) of 13.97 ​± ​7.70 compared to the non-VGX animal sensitivity of 29.74 ​± ​9.43, this difference was not statistically significant. Histologically, VGX animals showed thinner tibial cartilage and greater subchondral bone area than non-VGX animals (p ​= ​0.076; VGX: 0.80 ​± ​0.036 ​mm^2^; non-VGX: 0.736 ​± ​0.066 ​mm^2^). No group differences in systemic inflammation were observed at endpoint.

**Conclusions:**

VGX resulted in quicker onset of OA-related symptoms but remained unchanged at later timepoints. VGX also had thinner cartilage and abnormal bone remodeling than non-VGX. Overall, low vagal tone had mild effects on OA symptoms and joint remodeling, and not at the level seen in common OA comorbidities.

## 1 Introduction

Historically, osteoarthritis (OA) has been described as a localized and multifactorial disease hallmarked by pathological changes to the joint environment, such as articular cartilage degradation, abnormal bone remodeling, and inflammation [1]. As a result, treatment strategies have prioritized the local joint environment as a therapeutic target. Neuroimmune crosstalk between the OA joint environment and nervous system has recently expanded our perspective of OA pathophysiology [2,3]. For example, inflammation in the OA joint environment sensitizes joint nociceptors, which leads to peripheral sensitization [4,5]. Beyond the peripheral nervous system, sustained and increased firing of joint nociceptors can promote sensitization of the dorsal horn of the spinal cord, leading to central sensitization [[5], [6], [7], [8]]. The onset of peripheral and central sensitization promotes and maintains OA-related pain and may perturb autonomic nervous system (ANS) function [9]. A dysfunctional ANS has been suggested in multiple chronic musculoskeletal diseases [10] and even in a preclinical model of OA [11]. Due to the role of the ANS in regulating neuroimmune health, perturbations in its function could impact joint health. As a result, characterizing the impacts of a blunted ANS on OA pathophysiology is needed.

The ANS consists of two branches: the parasympathetic and sympathetic nervous systems. Both branches work in concert to regulate neuroimmune health via the neuroimmune axis [12]. Mechanistically, the parasympathetic nervous system coordinates peripheral immune homeostasis primarily through the vagus nerve. Specifically, efferent vagal signals modulate the cholinergic anti-inflammatory pathway [13], while afferent vagal signals modulate both the splanchnic anti-inflammatory pathway [14] and the hypothalamic-pituitary-adrenal (HPA) axis [15,16]. Overall, reduced vagal activity promotes a proinflammatory response in all three pathways and could exacerbate OA pathogenesis.

Apart from shared associations between neuroimmune health and vagal tone, the risk of developing OA may be greater with low vagal tone. For example, low vagal tone is associated with several OA comorbidities such as diabetes [17,18], hypertension [[19], [20], [21]], obesity [[22], [23], [24]] and risk factors such as physical inactivity [[25], [26], [27], [28]] and aging [20,29,30]. Additionally, increasing vagal tone via vagus nerve stimulation has alleviated symptoms in individuals with erosive hand OA [31] and has been suggested as a potential treatment strategy for OA [[32], [33], [34]]. Several mechanisms may be involved in the potential therapeutic effects of vagus nerve stimulation such as the activation of the cholinergic and splanchnic anti-inflammatory pathways [13,14], modulation of immunomodulatory cortisol levels [15,16], or disrupting the transmission of pain signaling [35]. Moreover, vagotomy (VGX) results in higher levels of systemic inflammation in mice than in their sham counterparts [36]; and, in preclinical models of rheumatoid arthritis, unilateral cervical VGX exacerbated arthritis scores [37]. Together, these shared associations suggest a potential pathophysiological link between vagal tone and OA pathogenesis. However, the degree of influence that low vagal tone may have on OA is unknown, especially when present in comorbidities and risk factors. Therefore, the present study sought to evaluate the direct influence of low vagal tone on OA pathogenesis, independent of comorbidity. Specifically, we tested the hypothesis that reducing vagal tone via VGX will accelerate OA-related symptoms and worsen joint damage in a rat model of chronic knee OA.

## 2 Materials and methods

### 2.1 Experimental design

As the effects of VGX have not been previously tested in an OA model, power calculations were conducted in the form of a sensitivity analysis (G∗Power, v. 3.1), which represent the smallest detectable effect size for the experimental design. For monthly gait measurements, specifying n ​= ​8 per group, α ​= ​0.05, β ​= ​0.2 (power ​= ​0.8), and four repeated measures yielded an effect size (f) of 0.28. For biweekly tactile sensitivity measures, the value of repeated measures was adjusted from four to seven, which yields an effect size (f) of 0.24. This effect size indicates that the global statistical model should detect a significant effect if the grouping factor or interaction explains 24–28% or more of the total variance. Due to the increased risk of humane endpoints in the VGX group, this group's sample size was increased to n ​= ​10. However, a sensitivity analysis was ultimately re-run using the resulting sample size (n ​= ​6 for non-VGX, n ​= ​9 for VGX) after dropout. This revealed a Cohen's f of 0.26 for bi-weekly and 0.31 for monthly behavioral measures, meaning a continuous measure must account for 26–31% of the global variation to be noted as a significant effect. At endpoint (week 12), serum, cerebral spinal fluid (CSF), knee joints, and ipsilateral dorsal root ganglia (L3–L5) were collected.

**Fig. 1:**
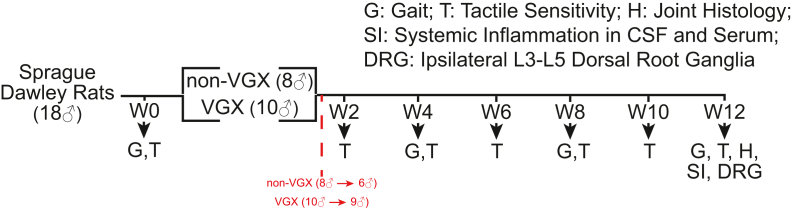
Summary of experimental design.

A random number generator was used to assign rats to two groups: VGX (n ​= ​10) or non-VGX (n ​= ​8). To manage postoperative pain, twice-daily buprenorphine (0.05 ​mg/kg) and once-daily meloxicam (5 ​mg/kg) were administered for 48 ​h beginning at time of surgery. Due to animals excessively grooming their stifle joint incision, a 14-day course of Baytril antibiotic water was applied to all animals to reduce the risk of infection. Immediately after surgeries, three animals reached a humane endpoint: two in the non-VGX group due to recurrent incisional dehiscence and one in the VGX group due to labored breathing. Fig. 1 provides a summary flow chart of the experimental design.

### 2.2 Animals

The University of Florida Institutional Animal Care and Use Committee approved all procedures. Male CD Sprague Dawley rats (12 weeks of age, weighing approx. 500 ​g) were obtained from Charles River (Wilmington, MA, USA) and acclimated for at least five days at the UF animal care facility. Animals were housed two per cage in a lighting-controlled environment (12-h light/dark cycle) with access to food and water *ad libitum.* In the case of animal dropout, the cage partner remained solo. As advised by UF veterinary staff, animals that grew to exceed 650 ​g were separated and housed individually to avoid adverse health impacts due to cage overcrowding. The number of animals transferred from paired to individual housing for each group is as follows: n ​= ​3 (VGX ​= ​2, non-VGX ​= ​1) immediately following surgery due to cage mate dropout, an additional n ​= ​6 (VGX ​= ​2, non-VGX ​= ​4) at week 4, and an additional n ​= ​4 (VGX ​= ​3, non-VGX ​= ​1) at week 8. Standard bedding and housing were used.

### 2.3 Surgical induction of knee osteoarthritis

Rats were first induced in anesthesia using 3% isoflurane in oxygen, and further maintained using 1.5–2% isoflurane in oxygen. First, the right knee was shaved and aseptically prepared by applying betadine and 70% ethanol to the shaved area three times, ending with a fourth application of betadine. For all surgeries, a 1–2 ​cm incision was made along the right stifle, slightly medial to the patella. Muscle was blunt dissected to expose the medial collateral ligament. To induce OA, the medial collateral ligament was transected, followed by a radial transection of the medial meniscus at its central aspect (MCLT ​+ ​MMT). Following transection, a simple interrupted suture technique was used to close muscle and a running subcuticular technique was used to close skin using sterile 4–0 polydioxanone sutures (13 ​mm 3/8 circle reverse-cutting needle).

### 2.4 Vagotomy surgery

The left cervical vagus nerve was transected to induce VGX using similar methods described by Caravaca et al. [36]. Immediately following knee surgery, rats remained anesthetized with isoflurane (1.5–2.0% in oxygen). The ventral cervical region of the rat was shaved and aseptically prepared using the same technique as for the stifle joint. With the surgeon blinded to group, a 1–2 ​cm midline incision was made along the ventral cervical area. Blunt dissection was performed to separate connective tissue and expose the two salivary glands. Salivary glands were then gently separated along their midline, exposing the sternomastoid and sternohyoid muscles. Blunt dissection was then performed left of the rat trachea to expose the left carotid sheath. The vagus nerve was then desheathed from the carotid sheath. To clarify, the surgeon was unaware of surgical group (VGX or non-VGX) from the start of incision until this point. At this point, the surgeon was told whether to transect (VGX) or not transect (non-VGX) the vagus nerve. For the non-VGX group, the incision was immediately closed. For the VGX group, sterile 6–0 nonabsorbable silk sutures were tied ½ cm apart on the vagus nerve and sealed onto the nerve using cyanoacrylate tissue adhesive (VetBond). The vagus nerve was transected between the two tied sutures using microscissors. For incision closure, a similar closing technique and suture material was used as described for OA induction.

### 2.5 Tactile sensitivity

The von Frey assay was used to measure tactile sensitivity, and 50% withdrawal thresholds were approximated using Chaplan's estimations [38]. One week before baseline recordings, animals were acclimated to wire-mesh floored cages for 30 ​min daily for three days. Then, for regular testing of tactile sensitivity, a series of von Frey filaments (Stoelting Co., Wood Dale, IL – item 58011) were applied to the plantar region of the hindfoot using an up-down approach. Data points for each time point correspond with one-day measures.

### 2.6 Spatiotemporal and dynamic gait recordings

One week before baseline recordings, animals were acclimated to our custom gait arena for 30 ​min daily for three days. Our custom gait arena consists of a 60 ​× ​5 ​× ​10-inch clear acrylic enclosure mounted to an aluminum frame. Inside the aluminum frame, a mirror angled at 45° beneath the arena floor allows for visualization of the rat's ventral and lateral views. Our lab's open-source software (https://github.com/OrthoBME/GAITORsuite.git), the GAITOR suite [39], was used to calculate various spatiotemporal variables (e.g., percentage stance time) using high-speed videography (500 frames/s, Phantom Micro Lab 320 camera) [[40], [41], [42], [43]]. Simultaneously with spatiotemporal data, peak vertical forces were measured using instrumented floor panels (9317B Kistler force links). The use of a high-speed camera allows us to detect more subtle OA-related gait compensations that are often imperceptible to the human eye [44]. Only trials where rats held normal walking velocities (25–75 ​cm/s) with two or more gait cycles were included in the final analysis. This resulted in a total of 821 gait trials for our final analysis.

### 2.7 Joint histopathology

At endpoint, knee joints were dissected and fixed in 10% neutral buffered formalin for 48 ​h at room temperature, followed by 14 days of decalcification in 10% formic acid. Then, knee joints underwent paraffin processing (Leica VIP6), and 10 ​μm frontal sections of the knee were collected. Three slides, representing the loading region, were stained with safranin-O and fast green. For synovitis assessment, an additional slide from the loading region was stained with hematoxylin and eosin. Finally, the tibial medial compartment of the joint was graded using open-source software that our lab has developed [45]. Briefly, a blinded grader using a MATLAB interface segmented the cartilage and bone from each image, then a MATLAB script calculated the total area of tibial cartilage, subchondral bone, and bone marrow space in the medial compartment of each section based on this segmentation. Additionally, three blind graders assigned a 0–6 score for each animal's histological image based on the OARSI histopathology grading system [46]. Similarly, a 0–9 score was provided for synovitis scoring using the method explained by Krenn et al. [47]. Specifically, the graders scored medial tibial cartilage and medial synovium, reaching a consensus score for every joint evaluated.

### 2.8 Multiplex Luminex assay

To not confound week 12 behavioral measures taken immediately prior to endpoints, animals were not fasted before CSF or serum collection. CSF was collected from the cisterna magna using the methods described here [48], then placed in cryovials and stored at −80 ​°C. A cardiac puncture was performed using an 18-gauge needle to collect blood. Then, blood was transferred to a serum separator tube, inverted ten times, allowed to clot upright at room temperature for 30 ​min, and moved to ice until centrifugation. Vacutainers were centrifuged for 15 ​min at 2200 ​rpm to extract serum and then stored in cryovials at −80 ​°C. A 12-plex Luminex assay was used to assess neuroinflammation (CSF) and systemic inflammation (serum). Specifically, this Luminex assay tested for the following targets: IL-1β, IL-6, IL-10, IL-12, IL-13, CXCL10, CCL2, CCL3, NGF, CCL5, TNF-α, and VEGF. Only markers with greater than 50% of samples above the lower detection limit were included in the statistical analysis. Our statistical analysis did not imputate results lower than the detection limit.

### 2.9 Reverse transcriptase-polymerase chain reaction (RT-PCR)

At endpoint, ipsilateral dorsal root ganglia (DRGs) were dissected from the lumbar (L3–L5) regions, immediately placed in RNAlater™ (Invitrogen) stabilization solution and stored at −20 ​°C. Contralateral DRGs were not collected. DRGs were then homogenized under chilled conditions using a sterile scalpel blade before being further homogenized using TRIzol® (Invitrogen) reagent. An RNeasy® mini kit (QIAGEN) was used to extract RNA, followed by its quantification using a NanoDrop™ (Thermo Fisher Scientific) spectrophotometer. Only samples having an A280/A260 ratio of 1.7 or greater were used to ensure high RNA purity; this resulted in the following sample sizes: n ​= ​5 for non-VGX and n ​= ​4 for VGX. Next, RNA was converted to cDNA using an iScript™ cDNA synthesis kit (Bio-Rad). Finally, PCR was performed using fast SYBR™ green mix (Applied Biosystems) and primers (Eurofins Genomics) for the following genes: *Gapdh*, *Trpv1*, *Nav1.7*, *Nav1.8*, *Cgrp*, *Ccl2*, *Cx3cl1*, and *Ngf*. The National Institutes of Health's Primer-Blast tool was utilized to design primers, and their sequences are provided in Table 1. To quantify the change in gene expression of VGX animals relative to non-VGX animals, the double change (ΔΔ) in threshold cycle (CT) method was applied. Due to an insufficient number of samples available for analysis (n ​= ​5 for non-VGX and n ​= ​4 for VGX), insufficient effect sizes were achieved, and statistical analysis was not performed.

**Table 1:** Gene primers used for RT-PCR.

Gene	Forward Primer Sequence (5′ to 3′)	Reverse Primer Sequence (5′ to 3′)
*Gapdh*	ACATGCCGCCTGGAGAAAC	AGCCCAGGATGCCCTTTAGT
*Trpv1*	GGGTTCACTCCTGACGGCAA	TTCTCCCTGAAACTCGGCCT
*Nav1.7*	CAGCCGCAGATAGCCGTCGT	GGGCGTCCGCAAAGTCAGAG
*Nav1.8*	CCGGTGGAAGCAGGAAGA	AGGAGCGGTGCAGCATGTA
*Ccrp*	ATGGGCTTTCTGAAGTTCTCC	GGGCTGCTTTCCAAGGTTGAC
*Ccl2*	ATGCAGTTAATGCCCCACTC	TTCCTTATTGGGGTCAGCAC
*Cx3cl1*	CTCCAGCCATCCAGCCATG	CATTTCGTCATGCCGAGGTG
*Ngf*	CGAAGGGGAGCGCATCG	GACATTACGCTATGCACCTCAG

### 2.10 Statistical analysis

Gait and tactile sensitivity measures were analyzed using a linear mixed effects model, where animal ID was a random factor and surgery and time were fixed factors. To verify normality, model residuals were plotted on a Quantile-Quantile plot where data points consistently aligned with the theoretically expected (normal) 45° line. Additionally, residuals were plotted against fitted values of the model where homoscedasticity (equal variance) was observed. If directed by a significant ANOVA result, a post-hoc comparison of least squares mean estimates was performed within a specific fixed effect, correcting for multiple comparisons using Tukey's HSD test. To obtain the point estimate (mean) and 95% confidence interval for temporal/spatial symmetry (comparison to 50%) and stance time imbalance (comparison to 0%) at each time point for each surgery group, we adopted the nonpaired least squares means method. Least squares means are also known as marginal means, which are adjusted averages at each time point and for each surgery group (e.g., VGX group at week 4) that account for the model's covariates, random effects, and the overall design of the experiment. Moreover, the Shapiro-Wilk test was used to verify normality for quantitative histology and multiplex Luminex assay results. When data were not normally distributed, a Kruskal-Wallis test was performed. In the case where data were normally distributed, the equal variance assumption of the data was further verified by performing an F-test. In normally distributed data with equal variance, a two-sample Student's t-test was completed; in normally distributed data where variance was not equal, a two-sample Welch's *t*-test was performed. Additionally, OARSI and synovitis scores were evaluated using a Kruskal-Wallis test. All statistical analyses were performed using RStudio (v. 4.0.2).

## 3 Results

### 3.1 VGX resulted in mild joint remodeling and thinner cartilage

**Fig. 2:**
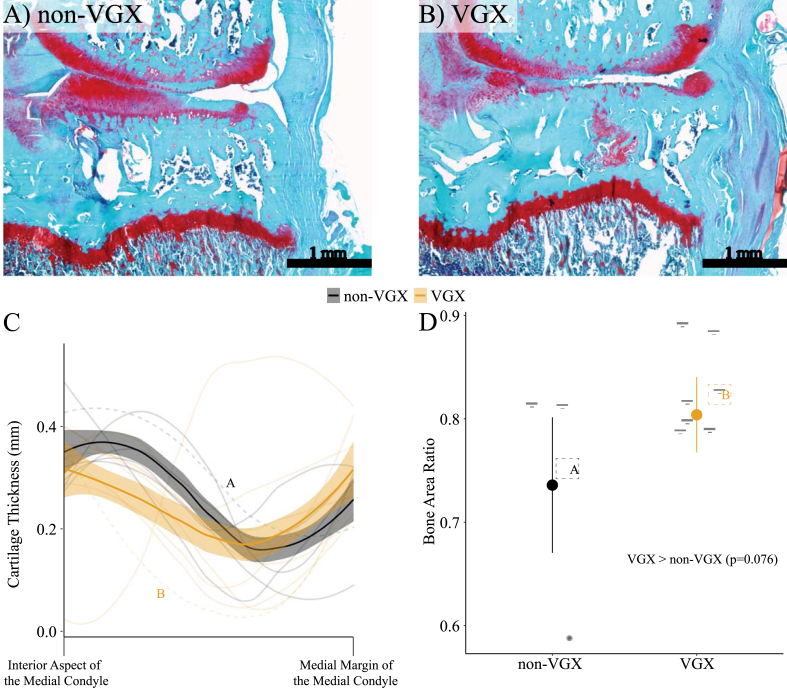
Representative histological sections (10 ​μm) showing A) non-VGX and B) VGX knee joints. Features of joint remodeling in the OA knee are shown by C) cartilage thickness along the medial tibial plateau and D) bone area ratio (the ratio of trabecular bone area to total epiphyseal area). For C, an x-axis value of 0% corresponds to the interior aspect (corresponding with the left sides of Fig. 2A and B) of the medial condyle, while a value of 100% indicates the medial margin (corresponding with the right sides of Fig. 2A and B) of the same medial condyle. The representative sections of figures A and B are represented in figure C by a dashed line with the label “A” and “B”, respectively. Similarly, the representative sections of figures A and B are represented in figure D by a dashed square with the label “A” and “B”, respectively. Error bands/bars represent a 95% confidence interval.

Representative images of the knee's medial compartment for both non-VGX and VGX animals are shown in Fig. 2A and B, respectively. For both groups, successful OA induction is represented by visual loss of red safranin-O staining and cartilage fissures/loss in the tibial cartilage. Features of joint remodeling quantified via GEKO [45] are shown in Fig. 2C and D. For VGX animals, cartilage was thinner along the interior aspect of the medial condyle compared to non-VGX animals, as indicated by the non-overlapping 95% confidence intervals (Fig. 2C). Cartilage thickness remained similar for both groups along all other regions of the medial condyle (Fig. 2C). In addition, bone area ratio (the ratio of trabecular bone area to total epiphyseal area) in the tibia of the OA knee tended to be greater in VGX animals (0.80 ± 0.036 mm^2^, 95% CI) than for non-VGX animals (0.736 ± 0.066 mm^2^, 95% CI), although this finding was nonsignificant (p = 0.076; Fig. 2D). Moreover, OARSI and synovitis scores revealed no group differences (Fig. S1). Representative histological images corresponding with low, middle, and high OARSI scores for each group are provided in Fig. S2.

### 3.2 VGX accelerated the onset of OA-related gait symptoms during early OA

**Fig. 3:**
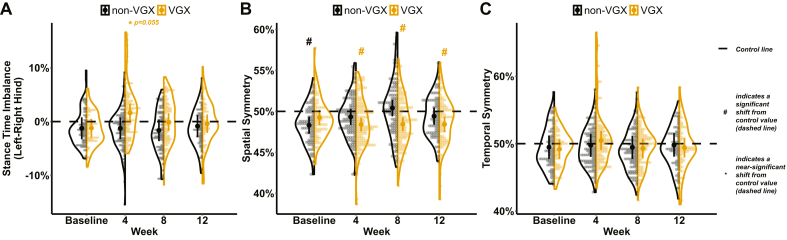
Gait balance and symmetry results for both groups. A) stance time imbalance of the hind limb (0% dashed line indicates equal stance times between the non-OA and OA limbs), B) spatial symmetry (50% dashed line indicates a foot strike of the OA limb placed halfway between two foot strikes of the non-OA limb), and C) temporal symmetry (50% dashed line indicates a foot strike of the OA limb occurring halfway between two foot strikes of the non-OA limb in time). A # indicates a significant shift (p ​< ​0.05), while a ∗ indicates a near-significant shift (0.05 ​< ​p ​< ​0.1) from the horizontal control (dashed) line. Error bars represent a 95% confidence interval.

**Fig. 4:**
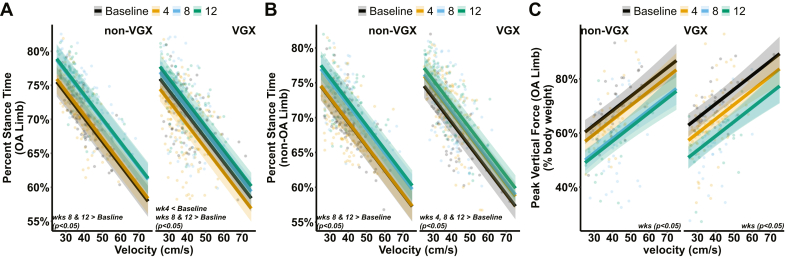
Individual stance times and peak vertical force for both groups. A) percent stance time of the OA limb, B) percent stance time of the non-OA limb, and C) peak vertical force as a percentage of body weight for the OA limb of both groups. Linear regression lines represent the predicted mixed-effect models for each group at a specific week. Individual points represent raw data. All error bands represent a 95% confidence interval.

At week 4, stance time imbalance — calculated as the stance time of the non-OA limb minus that of the OA limb for the same animal — was marginally greater than 0% (dashed line in Fig. 3A) for VGX animals at week 4 (p ​= ​0.055; 1.6% ​± ​1.6%, 95% CI), indicating that VGX animals shifted away from their OA limb to their non-OA limb during the weight-bearing phase of walking (i.e., stance) (Fig. 3A). These results are further corroborated when evaluating within-group differences in stance times specific to the OA and non-OA limbs (Fig. 4). Relative to baseline, VGX animals displayed lower stance times in the OA limb (Fig. 4A) and greater stance times in the non-OA limb (Fig. 4B) across all velocities at week 4 (p ​< ​0.05; varying mean and 95% CI across velocities). In non-VGX animals, stance times for both limbs remained unchanged from baseline at week 4. While our results emphasize within-group differences in VGX and not in the non-VGX group, a statistically significant group main effect was not observed.

Corresponding to the imbalanced stance times at week 4, VGX animals displayed a spatially asymmetric gait, while non-VGX animals remained spatially symmetric. Specifically, values for spatial symmetry — where 50% indicates that a right foot strike was placed halfway between two left foot strikes — were below 50% for weeks 4 (p ​< ​0.001; 48.4% ​± ​0.835%), 8 (p ​< ​0.001; 48.4% ​± ​0.854), and 12 (p ​< ​0.005; 48.4% ​± ​0.919%) in VGX animals while remaining close to 50% for non-VGX animals (Fig. 3B). These changes in spatial symmetry can be associated with a limp, where the position of the ipsilateral OA limb's foot strike is shifted closer to the previous contralateral limb's foot strike than the preceding one. Moreover, values for temporal symmetry — where 50% indicates that a right foot strike occurred halfway between two left foot strikes in time — remained near 50% for both groups across all weeks (Fig. 3C).

### 3.3 VGX did not alter gait characteristics at mid- and late-stage OA

Beyond week 4, stance times in the non-OA and OA limbs were similar in both groups. Specifically, stance times increased for both limbs from baseline values at week 8 (p ​< ​0.001; varying mean and 95% CI across velocities) and week 12 (p ​< ​0.001; varying mean and 95% CI across velocities) for both groups across all velocities; this is consistent with OA progression in rodents (Fig. 4A and B) [49]. In addition, stance times remained balanced for both groups at weeks 8 and 12 (Fig. 3A). Also, relative to baseline, peak vertical forces in the OA limb decreased over time within each group (p ​< ​0.05; varying mean and 95% CI across velocities), as expected throughout OA progression in rodents (Fig. 4C).

### 3.4 Evaluation of tactile sensitivity

**Fig. 5:**
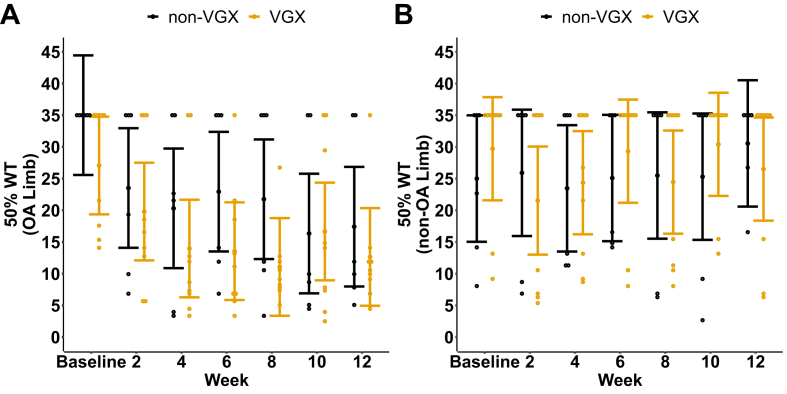
Tactile sensitivity measured via 50% paw withdrawal thresholds of the A) OA limb and B) non-OA limb across time for both groups. Individual points represent raw data, while filled points represent group means. All error bands represent a 95% confidence interval.

Tactile sensitivity is represented by the 50% withdrawal threshold measurement, as shown in Fig. 5. For the OA limb, both groups showed comparable decreases in their 50% withdrawal thresholds over time. Although not statistically significant, the relative drop in limb withdrawal threshold (indicative of heightened sensitivity) was larger in VGX animals than in non-VGX animals at weeks 4 (VGX ​= ​13.97 ​± ​7.70, 95%CI; non-VGX ​= ​29.74 ​± ​9.43, 95%CI), 6 (VGX ​= ​13.55 ​± ​7.70, 95%CI; non-VGX ​= ​22.94 ​± ​9.93, 95%CI), and 8 (VGX ​= ​13.97 ​± ​7.70, 95%CI, non-VGX ​= ​29.74 ​± ​9.43, 95%CI; Fig. 5A). For the contralateral non-OA limb, 50% paw withdrawal thresholds did not change for either group over time, as expected (Fig. 5B).

### 3.5 Evaluation of systemic inflammation at endpoint

Endpoint measures of systemic inflammatory markers in serum and CSF are shown in Figure S3. No group differences were detected in serum for CCL5, IL-10, IL-13, IL-12, CXCL10, CCL2, NGF, or CCL3. Moreover, IL-1β and TNF-α in serum were inconclusive due to the limited detection of samples. Although not shown here, no samples yielded detectable levels of VEGF and IL-6 in serum. In CSF, no group differences were detected in CXCL10, CCL2, and CCL5. Additionally, no samples yielded detectable levels of IL-1β, IL-6, IL-10, IL-12, IL-13, CCL3, NGF, TNF-α, and VEGF in CSF.

### 3.6 Evaluation of DRG gene expression at endpoint

**Fig. 6:**
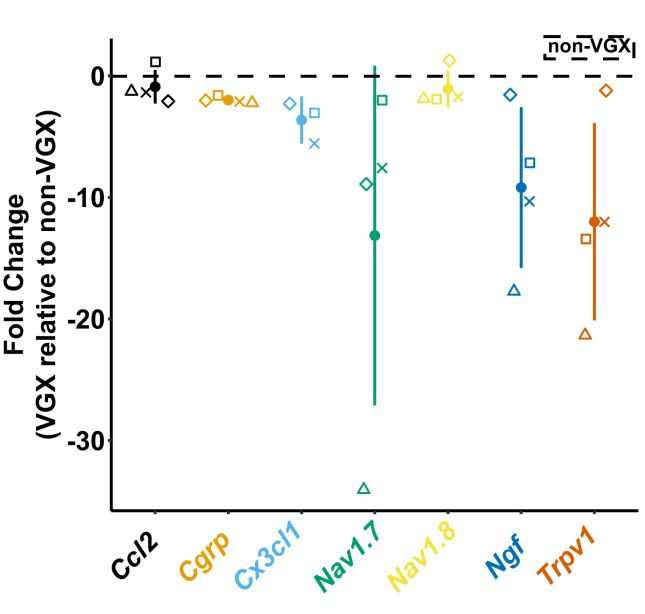
Changes in gene expression (2^−ΔΔCT^) for limb-innervating (lumbar 3–5) dorsal root ganglia ipsilateral to the OA limb. A value of zero indicates equal expression between both groups; a value greater or less than zero corresponds with upregulation or downregulation of genes relative to non-VGX animals, respectively. Individual shapes correspond to the same animal across all genes, while filled point represented the mean. Bars represent the 95% confidence interval. For CGRP, the 95% confidence interval was too small (±0.43) to be represented.

Unfortunately, the data available for assessing changes in DRG gene expression were limited due to insufficient RNA extraction from some animals. However, the limited data collected did indicate an observable decrease in *Cx3cl1*, *Nav1.7*, *Ngf*, and *Trpv1* expression in VGX animals relative to non-VGX animals at endpoint (Fig. 6). Due to the limited sample sizes obtained here, statistical analysis was not performed.

## 4 Discussion

Several reviews have highlighted the need to characterize crosstalk between the ANS and joint in OA pathophysiology [2,3,9]. Additionally, several clinical reports have explored the potential therapeutic effects of increasing vagal tone on OA [31,33,34]. However, no study has evaluated how low vagal tone influences disease progression in a rodent model of chronic OA. Here, we have begun to assess these relationships by evaluating the consequences of VGX on OA-related symptoms, joint structure, and systemic inflammation in a rat model of chronic knee OA.

Knee OA can cause altered gait patterns in rodents. For example, the MCLT ​+ ​MMT model produces a bilateral compensation to a unilateral meniscal tear in rodents, resulting in a shuffle-stepping gait [49,50]. Additionally, severe models of OA that result in greater sensitization of the nervous system are associated with antalgic gait compensations in rodents [8,43,50]. Since joint inflammation contributes to OA-related pain via peripheral [4,5] and central sensitization [[5], [6], [7], [8]], it is conceivable that the loss of peripheral inflammatory control via VGX could also result in similar antalgic gait compensations. Reviewing our gait results, VGX animals developed OA-related gait changes during early-stage OA (week 4) while not changing for non-VGX animals at mid-stage OA (week 8). The asymmetric and imbalanced gait that VGX animals displayed at week 4 is characteristic of an antalgic gait (i.e., limp), where animals load and unload their injured limb more quickly to avoid pain [50]. Interestingly, at earlier time points, paw withdrawal thresholds also tended to decrease for VGX animals than for non-VGX (though not significant). The coinciding nature of these findings may imply that low vagal tone contributes to the quicker onset of knee OA symptoms.

At later stages of OA progression (weeks 8 and 12), VGX and non-VGX animals displayed similar gait changes associated with the MCLT ​+ ​MMT model of OA [49,50]. The reversal of an antalgic gait profile in VGX animals at week 4 to a more similar gait profile shared between both groups at 8 and 12 weeks was unexpected; however, these changes suggest the physiological impact of VGX may have been temporary. Since the vagus nerve runs bilaterally across the cervical region, various opportunities exist to restore vagal crosstalk in response to unilateral ligation. Additionally, neurological compensatory mechanisms may have become activated and restored proper neuroimmune axis function in response to VGX [51,52]. It is also possible that VGX modified early OA symptoms by influencing post-surgical inflammation/pain inherent to the MCLT ​+ ​MMT model. Ultimately, cervical VGX acutely modified OA symptoms while having less of an effect at later time points. This emphasizes the importance of evaluating neuroimmune perturbations throughout different stages of OA pathogenesis, as shorter studies may miss the evolving nature of these relationships in later disease stages.

While lower vagal tone is associated with greater systemic and peripheral inflammation [53], our analysis of synovitis and systemic inflammation revealed no group differences. Despite previous evidence of VGX acutely increasing systemic inflammation [36], our measure was taken at endpoint and may not have captured the immediate effects of VGX such as alterations in immune-cell profiles [54], corticosterone levels [15,16], or proinflammatory cytokines [36]. By endpoint, it is possible that the systemic impacts of VGX were not as pronounced [51,52]. It is also important to note that our animals were not fasted before serum collection due to the confounding effects it may have presented on behavioral measures. Therefore, follow-up studies are needed to understand the relationship of behavioral changes and inflammation in the VGX-OA model.

Furthermore, our PCR results were conducted as an exploratory analysis meant to frame potential follow-up studies, and because our sample yield was small (n ​= ​4–5), we did not statistically analyze these data. However, an observable trend of lower expression levels for genes associated with peripheral sensitization (*Cx3cl1*, *Nav1.7*, *Ngf*, and *Trpv1*) was noted in the DRGs of VGX animals at endpoint. At the low n, mean estimates may be unstable as evidenced by the large confidence intervals observed for *Nav1.7*, *Ngf*, and *Trpv1*. Even so, the observed trend motivates further exploration of the effects of VGX on the peripheral nervous system in OA.

While OARSI and synovitis scores revealed cartilage damage and low-grade synovitis for both groups, they did not reveal group differences. This may be attributed to the inherent challenge of qualitatively detecting the subtle effects of VGX, especially as it relates to the high variability observed in our OARSI scores. However, our quantitative histopathology approach which considered spatial changes across cartilage and subchondral bone remodeling revealed mild group differences. Here, VGX animals showed thinner cartilage and some mild evidence of subchondral bone sclerosis compared to non-VGX animals. Although the mechanistic drivers of these changes were not explored, it is possible that VGX shifted the homeostasis of local ANS mechanisms at the joint. For example, crosstalk between the vagus nerve and the sympathetic nervous system may influence bone remodeling [55], joint inflammation [56,57], and chondrocyte metabolism [58]. Moreover, thinner cartilage and greater subchondral bone sclerosis have been observed in hypertensive OA rats with a dysregulated ANS [59]. While our results suggest vagal tone as a potentially shared root, the observed shifts in this study were less substantial than those seen with hypertension; thus, while low vagal tone may contribute to OA progression in some comorbid conditions, it is unlikely to be the primary factor. It's also important to note that our results are limited to male rats, and follow-up studies using female rats are needed to form a more complete understanding.

In conclusion, the primary goal of this work was to evaluate the direct role of vagal tone on OA pathogenesis. While VGX accelerated onset of OA symptoms, it did not worsen symptoms at later timepoints. Also, VGX contributed to more abnormal bone remodeling and thinner cartilage. Together, the effects of VGX on OA were mild at most. Future studies should investigate additional mechanisms within the brain-joint axis (e.g., sympathetic activity, HPA axis), as low vagal tone independently is unlikely the primary driver of disease in OA comorbidities.

## Author contributions

CJC contributed to the experimental design, animal surgeries, data collection, data analysis and interpretation, and the drafting and revision of the manuscript. TDY contributed to the experimental design, animal surgeries, and manuscript revision. JLG contributed to the histological analysis, data interpretation, and manuscript revision. KDA contributed to the experimental design, animal surgeries, data analysis and interpretation, and the drafting and revision of the manuscript.

## Funding

The 10.13039/100000069National Institute of Arthritis and Musculoskeletal and Skin Diseases of the 10.13039/100000002National Institutes of Health (10.13039/100000069NIAMS/10.13039/100000002NIH) supported this study under award numbers R01AR071431 and R01AR071431-03S1, and by a graduate student fellowship from the Herbert Wertheim College of Engineering and J. Crayton Pruitt Family Department of Biomedical Engineering at the University of Florida. Beyond providing funds, these sources did not participate in data collection, analysis, interpretation, or decision to submit this publication.

## Availability of data and materials

The data that support the findings of this study are available from the corresponding author, KDA, upon reasonable request.

## Conflicts of interest

CJC, TDY, and JLG declare that they have no competing interests. KDA is an associate editor for osteoarthritis and cartilage.
