# Supplementary material for: Vagotomy accelerates the onset of symptoms during early disease progression and worsens joint-level pathogenesis in a male rat model of chronic knee osteoarthritis
Source: Osteoarthr Cartil Open. 2024 Apr 8;6(2):100467. doi: 10.1016/j.ocarto.2024.100467 (PMC11035058; doi:10.1016/j.ocarto.2024.100467)
Supplement: Multimedia component 1 [file mmc1.docx]

**Supplementary Materials**

**Supplemental Fig. 1**

**
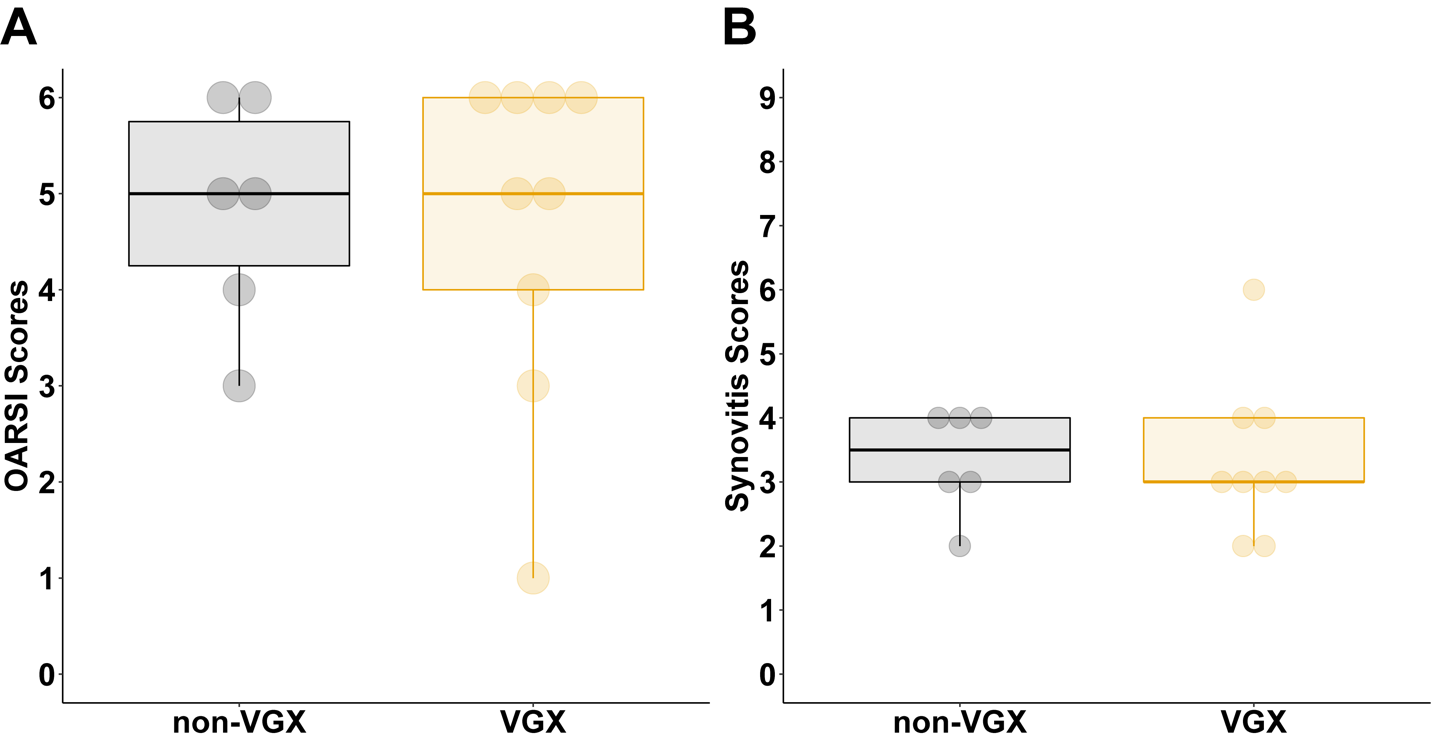
**

**Fig. S1** A) OARSI scores used to grade the medial tibial region of the OA limb and B) synovitis scores for medial synovium of the OA limb. Data points represent scores for individual animals. Box plots represent the median and interquartile range, while lines represent the range of the data.

**Supplemental Fig. 2

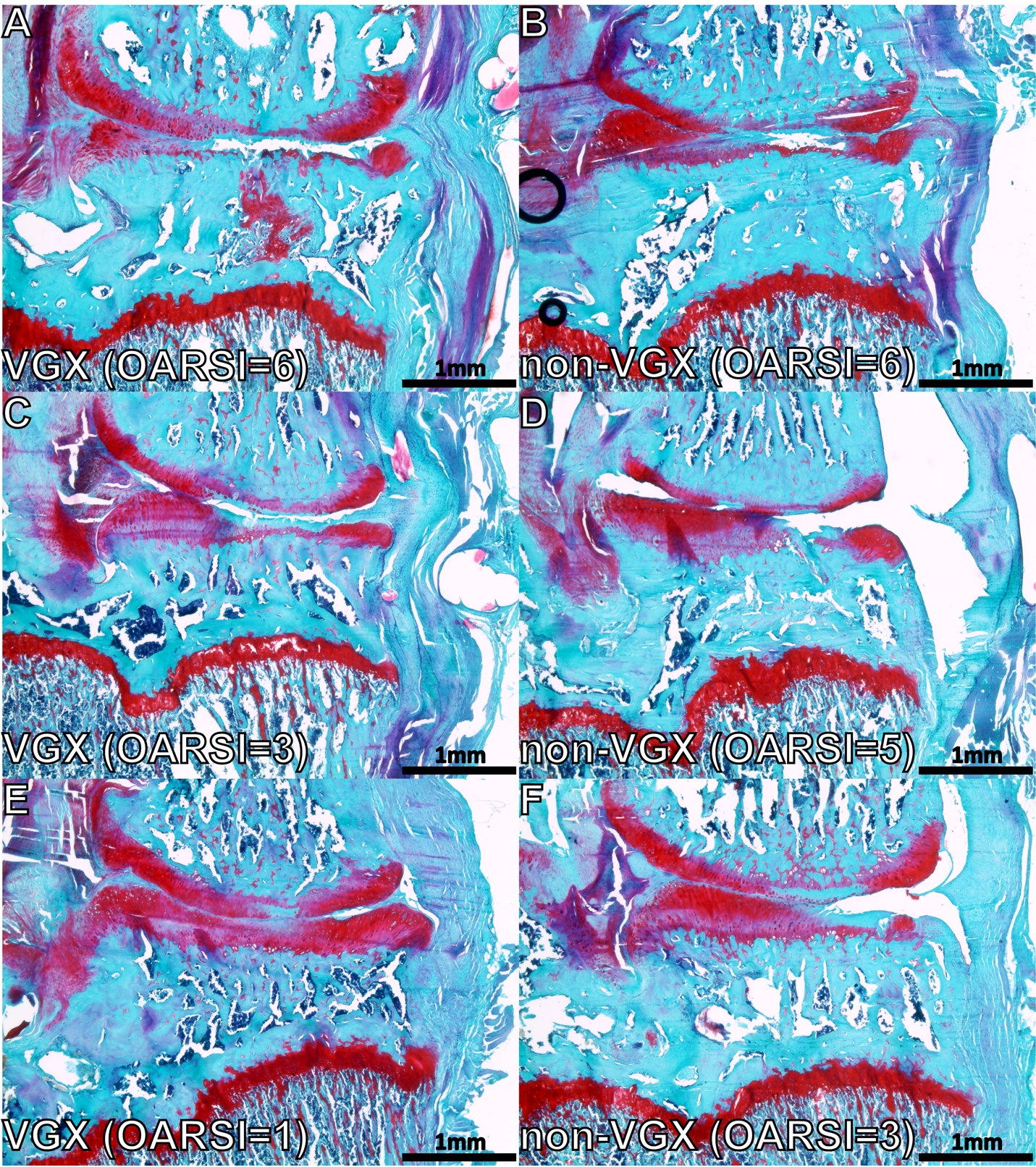
**

**Fig. S2** Representative histological images of the OA joint’s medial compartment showing low, middle, and high OARSI scores for both groups. For VGX animals, images are shown representing A) a high score of 6, C) a middle score of 3, and E) a low score of 1. For non-VGX animals, images are shown representing B) a high score of 6, D) a middle score of 5, and a F) a low score of 3.

**Supplemental Fig. 3**


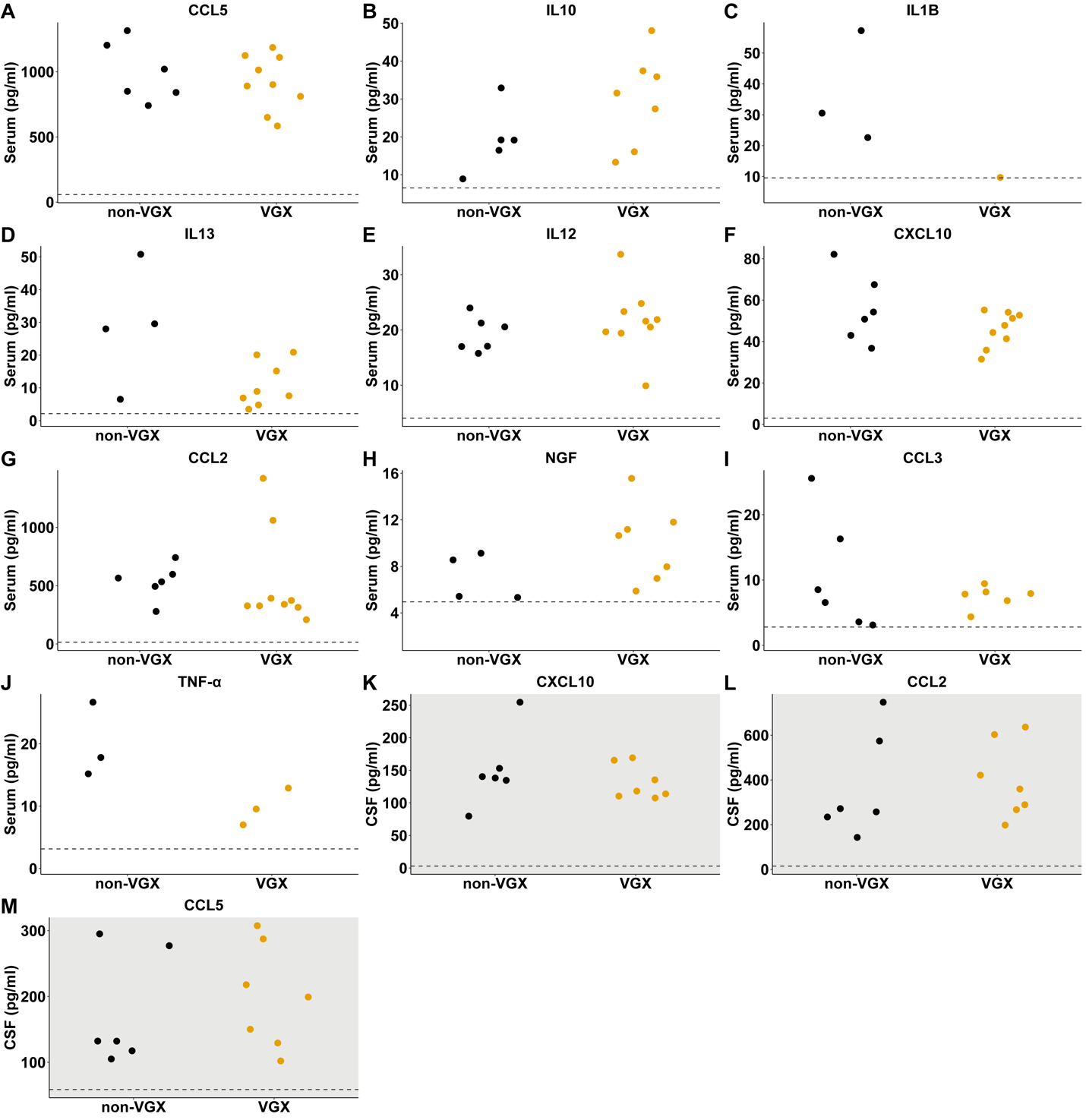


**Fig. S3** Endpoint measurements of systemic inflammation in serum and CSF (shaded gray) for VGX and non-VGX animals. Horizontal dashed lines indicate the lower limit of detection.
